# Chemical stability and compatibility of acetaminophen injection with six common opioid drugs: implications for clinical use

**DOI:** 10.1590/1414-431X2026e15263

**Published:** 2026-04-17

**Authors:** Weilin Huang, Shuyang He, Miao Zhao, Shunyuan Hong, Junhong Xu, Limin Chen, Xianping Wu

**Affiliations:** 1 Department of Anesthesiology, Shunde Hospital of Guangzhou University of Chinese Medicine, Foshan, Guangdong Province, China; 2 Institute of Anesthesiology, Guangzhou University of Chinese Medicine, Guangzhou, Guangdong Province, China; 3 Department of Pharmacy, Shunde Hospital of Guangzhou University of Chinese Medicine, Foshan, Guangdong Province, China

**Keywords:** Acetaminophen injection, Opioid drugs, Drug compatibility, Chemical stability, High-performance liquid chromatography

## Abstract

Despite the clinical importance of combining acetaminophen and opioids in multimodal analgesia, their compatibility has not been determined. This study aimed to investigate the stability and feasibility of mixing acetaminophen injection with six commonly used opioid drugs. Acetaminophen injection was mixed with each of the six opioid drugs at room temperature (25°C). Changes in appearance, pH, and acetaminophen concentrations were monitored at different time intervals to evaluate compatibility. Compatibility was defined as no visible changes, pH variation <1.0, and acetaminophen concentration ≥90% of baseline. The results indicated that acetaminophen injection, when mixed with six commonly used opioid drugs, remained stable for up to 48 h at room temperature. No significant changes were observed in the appearance of the mixtures, which remained clear and free of precipitation. The pH of the mixtures fluctuated by less than 1.0 unit, and the acetaminophen concentration remained above 90% of the baseline value, with a variation of less than 10%. Acetaminophen injection was compatible with morphine, sufentanil, hydromorphone, pentazocine, butorphanol, and nalbuphine for at least 48 h at 25°C, supporting their co-use in patient-controlled intravenous analgesia. Further studies should define pharmacokinetics and adverse effects of these combinations.

## Introduction

Acetaminophen (paracetamol) is a widely used analgesic and antipyretic, commonly administered via oral, rectal, or intravenous formulations (1-5). Acetaminophen is efficacious, acts rapidly, and has minimal effects on the gastrointestinal tract and coagulation mechanisms even in nursing mothers and children (6-8). While its mechanism of action has been traditionally attributed to the inhibition of cyclooxygenase enzymes, recent research suggests that acetaminophen’s primary analgesic effects may be mediated through its metabolites binding to transient receptor potential vanilloid receptors and cannabinoid 1 receptors in the brain (9). Despite its efficacy, acetaminophen exhibits instability in aqueous solutions, often undergoing hydrolysis to form aminophenol, which can degrade further into quinoneimine. This instability presents a potential risk in intravenous formulations, particularly when mixed with other medications.

Mannitol helps prevent the degradation of active pharmaceutical ingredients (APIs) caused by ice crystal formation during the lyophilization process, thereby enhancing the drug's stability (10). In the case of acetaminophen and mannitol injection, the inclusion of mannitol in the formulation helps mitigate this degradation by maintaining a stable pH range between 5.5 and 6.5.

In clinical practice, acetaminophen is frequently combined with opioid analgesics in multimodal pain management protocols (11-13). This combination aims to enhance analgesic effects while reducing opioid consumption, a key strategy in opioid-sparing approaches (14,15). However, concerns about the compatibility of acetaminophen with various drugs remain, particularly in intravenous formulations where incompatibilities could lead to reduced efficacy or harmful interactions (16,17).

Similar research on the Y-site compatibility of acetaminophen with other central nervous system medications has been reported by Hanifah et al. (18), who demonstrated variable compatibility depending on co-administered drugs. Moreover, a scoping review by Hanifah et al. (19) emphasized that standardized compatibility testing helps to establish reproducible combination formulas, thereby improving the safety of injectable analgesic regimens in clinical practice. Despite the clinical importance of acetaminophen-opioid combinations, data on their chemical compatibility and stability are sparse. This study aimed to fill this gap by evaluating the stability and compatibility of acetaminophen with six commonly used opioid drugs (morphine, sufentanil, hydromorphone, pentazocine, butorphanol, and nalbuphine), providing critical information for their safe use in intravenous analgesia.

## Material and Methods

### Instruments

An UltiMate3000 high-performance liquid chromatography (HPLC) system (including a column oven, an autosampler, a fluorescence detector (Thermo Fisher Scientific, USA)); Nano-500 microspectrophotometer (Alpha Biotech Equipment Co, Ltd., China); KH3200B ultrasonic cleaner (Hecr Instrument Co., Ltd., China); FA2004A analytical balance with a resolution of 1/10000 (Jingtian Electronic Instrument Co., Ltd., China); and GREENER pen-type pH tester (Greenforest Tools Co., Ltd., China) were used in this study.

### Drugs and Reagents

The following drugs and reagents were used: 1) Acetaminophen and mannitol injection (for intravenous use, concentration: 10 mg/mL; specification: 50 mL: 500 mg; batch number: 230612DE; Jiangsu Hengrui Medicine Co., Ltd., China); 2) Morphine sulfate injection (for intravenous use, concentration: 10 mg/mL; specification: 1 mL: 10 mg; batch number: 231006; Northeast Pharmaceutical Group Shenyang First Pharmaceutical Co., Ltd., China); 3) Sufentanil citrate injection (for intravenous use, concentration: 50 µg/mL; specification: 1 mL: 50 µg; batch number: 31A102112; Yichang Renfu Pharmaceutical Co., Ltd., China); 4) Hydromorphone hydrochloride injection (for intravenous use, concentration: 1 mg/mL; specification: 2 mL: 2 mg; batch number: 33A050812; Yichang Renfu Pharmaceutical Co., Ltd.); 5) Pentazocine injection (for intravenous use, concentration: 30 mg/mL; specification: 1 mL: 30 mg; batch number: G2309063; CSPC Shuanghe Pharmaceutical Co., Ltd., China); 6) Butorphanol tartrate injection (for intravenous use, concentration: 2 mg/mL; specification: 2 mL: 4 mg; batch number: 231020BP; Jiangsu Hengrui Medicine Co., Ltd.); 7) Nalbuphine hydrochloride injection (for intravenous use, concentration: 10 mg/mL; specification: 2 mL: 20 mg; batch number: 23061761; Yangtze River Pharmaceutical Group Co., Ltd., China); 8) Methanol (chromatographically pure; batch number: 20240327; Tianjin Kemiou Chemical Reagent Co., Ltd., China); 9) Ammonium acetate (chromatographically pure; batch number: 20230701; Tianjin Kemiou Chemical Reagent Co., Ltd.); 10) Acetylaminophenol analysis standard reagent (reagent purity >99.5%; batch number: RH603326; Shanghai Lyn Technology Development Co., Ltd., China); 11) Ultrapure water.

### Chromatographic conditions

The chromatographic conditions used for acetaminophen analysis were as follows: 1) Chromatographic column: Bizcomr-XY (4.6×300 mm, 5 μm); 2) Mobile phase A: 0.05 mol/L ammonium acetate; 3) Mobile phase B: Methanol; 3) Gradient elution procedure: At 0 min, the mobile phase composition was 85% ammonium acetate and 15% methanol, with a flow rate of 0.8 mL/min. From 15 to 20 min, the mobile phase composition remained the same (85% ammonium acetate and 15% methanol), but the flow rate was increased to 1.0 mL/min and then returned to 0.8 mL/min at 20 min; 4) Column temperature: 30°C; 5) Detection wavelength: 245 nm; 6) Injection volume: 20 μL.

### Solution preparation

The standard reference control solution was 100 mL of acetaminophen and mannitol injection (containing 1000 mg of acetaminophen) + 20 mL of 0.9% sodium chloride. Mixture solutions were the following: Solution I: acetaminophen injection 100 mL + morphine injection 20 mL made up to a total volume of 120 mL; Solution II: acetaminophen injection 100 mL + sufentanil citrate injection 0.5 mL + 0.9% sodium chloride 19.5 mL made up to a total volume of 120 mL; Solution III: acetaminophen injection 100 mL + hydromorphone injection 10 mL + 0.9% sodium chloride 10 mL to a total volume of 120 mL; Solution IV: acetaminophen injection 100 mL + pentazocine injection 3 mL + 0.9% sodium chloride 17 mL made up to a total volume of 120 mL; Solution V: acetaminophen injection 100 mL + butorphanol tartrate injection 2 mL + 0.9% sodium chloride 18 mL to a total volume of 120 mL. Solution VI: acetaminophen injection 100 mL + nalbuphine hydrochloride injection 8 mL + 0.9% sodium chloride 12 mL to a total volume of 120 mL. Since the standard volume of postoperative patient-controlled analgesia (PCA) pumps typically ranges from 100 to 150 mL, we standardized the volume of all prepared solutions to 120 mL to simulate the capacity conditions in PCA pumps. The standard reference solution and the mixed solutions were stored in a drug stability testing chamber maintained at a constant temperature of 25°C and humidity of 80%. In the mixture solution of acetaminophen and morphine injection, the morphine dose corresponded to the commonly used therapeutic dose for postoperative intravenous continuous analgesia. In the mixture solutions of acetaminophen and other opioid drugs, the doses of the opioids were converted to equivalent doses based on the morphine dose (20 mL, 200 mg).

### Methodological experiments

#### Dilution of solutions

Two milliliters of the mixed solution was measured precisely and transferred to a 50-mL beaker. An equal volume of ultrapure water was added and the solution was mixed thoroughly. The solution was then diluted with water in a 1:1 ratio to yield a 2× diluted solution. These steps were repeated until the solution had been diluted 32 times (corresponding to acetaminophen standard concentration of 130 µg/mL). Each dilution step was carefully recorded.

#### Limit of detection and limit of quantification

Using the slope (2.538) of the HPLC calibration curve and the standard deviation of the intercept (σ=1.12), the limit of detection (LOD) was calculated as 1.46 µg/mL, and the limit of quantification (LOQ) was found to be 4.42 µg/mL. Experimental verification confirmed that the LOQ concentration range (4.0625-5.0 µg/mL) achieved a signal-to-noise ratio (S/N) ≥10 and exhibited a relative standard deviation (RSD) of peak areas of 2.1%, thus meeting the ICH Q2(R1) guideline criteria (RSD 5%). These results demonstrated the reliability of detection and the quantification capabilities of the method at low concentrations.

#### Specificity

**Figure 1:**
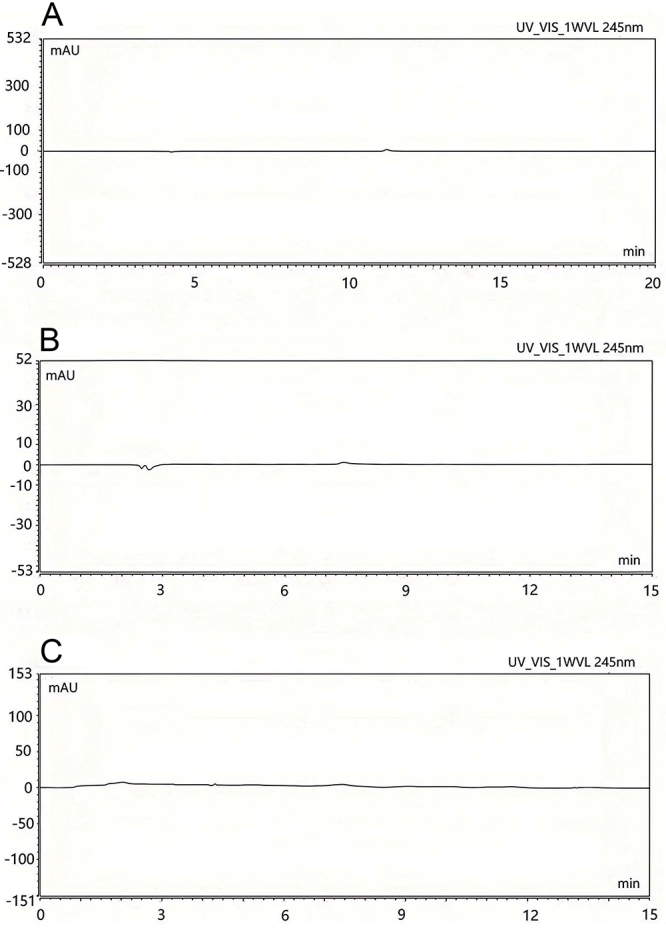
High-performance liquid chromatography chromatograms validating excipient interference. **A** ultrapure water; **B**, 0.9 sodium chloride; **C**, mannitol.

Ultrapure water was used as the blank control. To eliminate the interference caused by ultrapure water, mannitol, and sodium chloride mixed with acetaminophen injection, samples of ultrapure water, mannitol, and 0.9% sodium chloride solution were injected and measured under the chromatographic conditions. The results showed no interference, indicating good specificity for the method (Figure 1).

**Figure 2:**
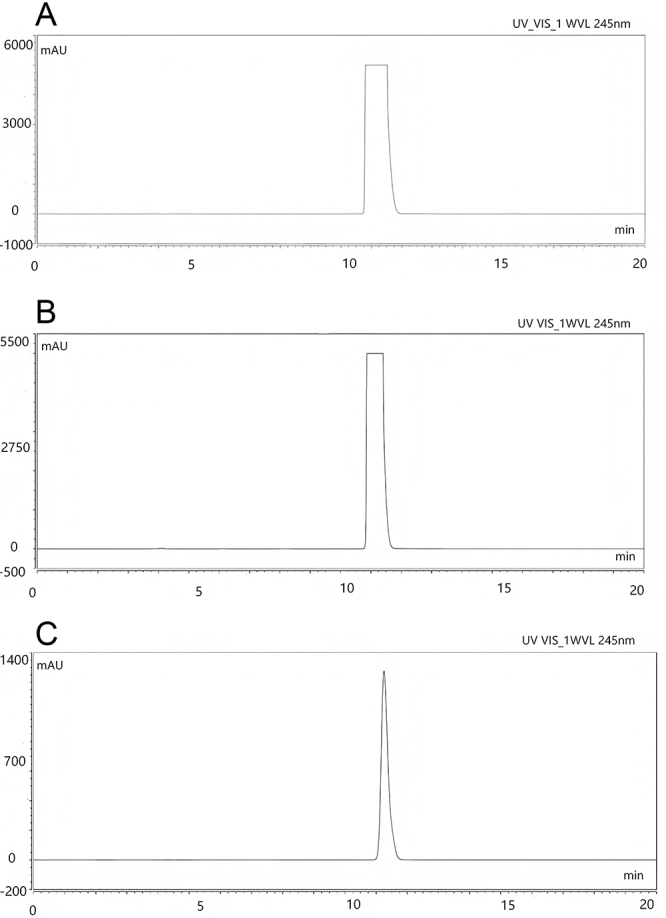
Comparative high-performance liquid chromatography of acetaminophen analytical standard reagent (**A**) and acetaminophen standard solutions at different dilution levels: (**B**) stock solution and (**C**) 32-fold dilution.

The acetaminophen analytical standard reagent, acetaminophen standard reference (stock solution), and acetaminophen standard reference solution (32-fold dilution) were each used for content determination utilizing the chromatographic conditions. The chromatograms are shown in Figure 2. The acetaminophen standard reference solution (32-fold dilution) and acetaminophen standard reference (stock solution) showed corresponding chromatographic peaks at the same retention time as the acetaminophen analytical standard reagent. All the chromatograms showed single, well-defined peaks.

**Figure 3:**
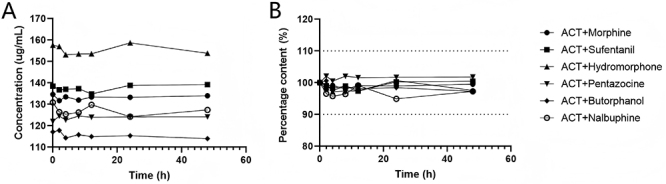
Changes in concentration (**A**) and percentage content (**B**) of various acetaminophen (ACT)-opioid admixture solutions over 48 h.

#### Linearity

Appropriate volumes of standard reference solutions were measured and diluted with ultrapure water to prepare a series of standard solutions with acetaminophen concentrations of 4.0625, 8.125, 16.25, 32.5, 65, and 130 µg/mL. Each series of standard solutions was then injected sequentially according to the chromatographic conditions, and the chromatograms and peak areas were recorded.

Linear regression was performed with the peak area (y) plotted against the concentration of the standard solution (×) to obtain the equation y=2.538× + 3.4587 (R^2^=0.9997, n=6), indicating a good linear relationship within the concentration range of 4.0625-130 µg/mL for acetaminophen. The linearity (R^2^=0.9997) met the standards of the ICH Q2(R1) guidelines for analytical method validation.

#### Accuracy

Six consecutive injections of the standard reference solutions were performed following the chromatographic conditions, and the peak areas were recorded. Acetaminophen concentration was calculated from the standard curve, and the RSDs were determined. The concentrations of acetaminophen from the six measurements were 143.384, 149.19, 150.208, 149.701, 150.177, and 150.050 µg/mL, with an RSD of 1.78%, indicating the good precision of the method and meeting the requirements of the assay.

#### Precision

The precision of the HPLC method was evaluated by determining both intra-day and inter-day precision. For intra-day precision, three different concentration levels of acetaminophen (4.0625, 32.5, and 130 µg/mL) were tested in triplicate on the same day. The RSDs of the peak areas for each concentration were calculated, which were found to be 1.2, 1.5, and 1.1%, respectively. These values indicate the method's good reproducibility within a single day. To assess inter-day precision, the same concentrations of acetaminophen (4.0625, 32.5, and 130 µg/mL) were measured on three consecutive days. Each concentration was injected in triplicate per day. The RSDs for the peak areas of acetaminophen across the three days were 1.6, 1.8, and 1.3%, respectively. These results confirm that the method demonstrates stable performance over multiple days, ensuring reliable results across time.

#### Fortified recovery rates

One milliliter each of solutions I-VI with known contents were accurately measured, and each solution was placed in a 2-mL volumetric flask. Six parallel replicates were performed. Next, a certain mass concentration of the standard control solution at 1 mL each was accurately added. After mixing, the samples were analyzed and the contents were determined.

The recovery rates of solutions I-VI ranged from 96.81 to 104.06%, which are within the standard range for fortified recovery experiments. The RSD values for the fortified recovery experiments of mixed solutions I-VI were 1.66, 1.41, 1.72, 1.52, 1.18, and 1.38%. The detailed results are reported in Table 1.

**Table 1:** Mean fortified recovery rates of acetaminophen mixed with six opioid drugs (n=6).

Mixture solution	Mean recovery	RSD	n
Acetaminophen + Morphine	98.9±1.7%	1.66%	6
Acetaminophen + Sufentanil	99.9±1.4%	1.41%	6
Acetaminophen + Hydromorphone	101.9±1.7%	1.72%	6
Acetaminophen + Pentazocine	100.9±1.5%	1.52%	6
Acetaminophen + Butorphanol	100.1±1.2%	1.18%	6
Acetaminophen + Nalbuphine	99.9±1.4%	1.38%	6

Data are reported as means±RSD (%). RSD: relative standard deviation.

### Stability tests

#### Appearance

Solutions I-VI were each sealed in a clean 50-mL beaker and placed against a standard black-and-white background. The color, clarity, and the presence of precipitation or turbidity were observed at 0, 2, 4, 8, 12, 24, and 48 h at room temperature by visual observation.

#### pH

The pH of the solutions was measured using a pen-type pH tester. The pH meter was calibrated with standard buffer solutions (pH 4.0, 7.0, 10.0) before each measurement. pH readings were taken at 0, 2, 4, 8, 12, 24, and 48 h. After each measurement, the pH pen was rinsed with ultrapure water to avoid cross-contamination.

#### Assay of pharmaceuticals

I-VI mixture solutions were placed at room temperature and sampled for analysis at the 0, 2, 4, 8, 12, 24, and 48 h time points, and the peak areas were recorded. The acetaminophen concentration values were calculated based on the regression equation described in “Linearity”, with the acetaminophen content of each mixture at 0 h at room temperature set as 100%.

#### Compatibility assessment methods

The compatibility evaluation in this study was designed as a physicochemical compatibility test under simulated Y-site and PCA conditions. Three parameters were used to determine compatibility: a) visual inspection; b) pH measurement; and c) acetaminophen concentration by HPLC.

The justification for this testing strategy follows established methodologies for evaluating intravenous drug compatibility (20). Incompatibility was defined as any of the following: a) visible precipitation, color change, or turbidity; b) pH change exceeding 1.0 unit from baseline; or c) a change of more than 10% in acetaminophen concentration from initial value.

When all three parameters remained within acceptable ranges, the combination was considered compatible. If any one of the three showed deviation beyond the specified limits, the mixture was considered incompatible. This multi-dimensional approach allows comprehensive assessment of both physical and chemical stability.

### Statistical analysis

Statistical analysis was performed using SPSS 26.0 (IBM Corp., USA). Data are reported as means±SD. Repeated measures ANOVA was applied to evaluate changes in acetaminophen concentration across different time points (0, 2, 4, 8, 12, 24, and 48 h) for each admixture. A P value <0.05 was considered statistically significant.

## Results

### Appearance and pH

Under room temperature (25°C) conditions, the acetaminophen injection solution, when mixed with the solutions of morphine hydrochloride, sufentanil citrate, hydromorphone hydrochloride, pentazocine, butorphanol, or nalbuphine hydrochloride, remained colorless and transparent with no precipitation within 48 h.

All mixed solutions maintained a pH range of 4.05-5.89, which is within the acceptable pH range for human tolerance (pH 4-9), with a fluctuation of less than 1.0 unit during the 48-h period (Table 2).

**Table 2:** pH value of acetaminophen mixed with six opioid drugs within 48 h.

Mixture solution	0 h	2 h	4 h	8 h	12 h	24 h	48 h
Acetaminophen + Morphine	5.02	5.51	5.68	5.56	5.47	5.50	5.77
Acetaminophen + Sufentanil	5.55	5.66	5.88	5.85	5.89	5.62	5.70
Acetaminophen + Hydromorphone	4.30	4.32	4.35	4.38	4.37	4.41	4.44
Acetaminophen + Pentazocine	4.05	4.26	4.30	4.12	4.30	4.21	4.30
Acetaminophen + Butorphanol	4.82	5.01	5.07	5.07	5.10	4.93	4.99
Acetaminophen + Nalbuphine	4.25	4.32	4.33	4.39	4.32	4.34	4.39
Acetaminophen (stock solution)	5.70	5.67	5.66	5.72	5.67	5.73	5.79

### Assay of pharmaceuticals

The acetaminophen percentage content in solutions I-VI at other time points changed by less than 10%, with RSD values below 2%. In Figure 3B, the acceptable range is represented by the area between the two dashed lines on the y-axis. Figure 3A shows the changes in acetaminophen concentration across the solutions I-VI. The results shown in Table 3 indicate that, within 48 h at room temperature, the acetaminophen injection solution exhibited relatively stable content when mixed with the six commonly used opioid drugs, meeting the pharmacopoeia stability standards (±10%). No significant degradation in acetaminophen concentration occurred within 48 h for any of the six admixture solutions (P=0.12).

**Table 3:** Determination of the concentration and percentage content of mixture solutions.

Mixture Solution	Test items	0 h	2 h	4 h	8 h	12 h	24 h	48 h
Acetaminophen + Morphine	Acetaminophen concentration (µg/mL)	134.613	131.709	133.472	131.960	133.395	133.259	133.969
	Percentage content	100.00%	97.84%	99.15%	98.03%	99.10%	98.99%	99.52%
	RSD	0.78%						
Acetaminophen +Sufentanil	Acetaminophen concentration (µg/mL)	138.497	136.758	137.035	137.250	134.843	138.883	139.162
	Percentage content	100.00%	99.00%	98.24%	98.94%	97.36%	100.28%	100.48%
	RSD	1.17%						
Acetaminophen + Hydromorphone	Acetaminophen concentration (µg/mL)	157.599	156.955	153.144	153.490	153.585	158.662	153.819
	Percentage content	100.00%	99.59%	97.17%	97.39%	97.45%	100.67%	97.60%
	RSD	1.50%						
Acetaminophen + Pentazocine	Acetaminophen concentration (µg/mL)	122.012	124.532	122.658	124.527	123.995	124.107	124.198
	Percentage content	100.00%	102.07%	100.53%	102.06%	101.63%	101.72%	101.79%
	RSD	0.80%						
Acetaminophen+ Butorphanol	Acetaminophen concentration (µg/mL)	117.124	117.818	114.348	115.780	114.906	115.308	113.962
	Percentage content	100.00%	100.61%	97.63%	98.85%	98.11%	98.45%	97.30%
	RSD	1.23%						
Acetaminophen + Nalbuphine	Acetaminophen concentration (µg/mL)	130.889	126.386	125.359	126.203	129.773	124.218	127.352
	Percentage content	100.00%	96.56%	95.77%	96.42%	99.15%	94.90%	97.30%
	RSD	1.88%						

RSD: relative standard deviation.

## Discussion

Acetaminophen injection, marketed in the USA as Ofirmev, is an antipyretic and analgesic administered by intravenous infusion and indicated for adults and children (21-23). Acetaminophen injection has been commercially available since 2001 in many countries in addition to the USA (24). In recent years, the American Society of Regional Anesthesia and Pain Medicine has strongly recommended the routine use of acetaminophen in postoperative multimodal analgesia (25,26). The combination of acetaminophen and morphine exhibits synergistic analgesic effects, reducing opioid consumption while improving patient satisfaction (27).

Our study demonstrated that, under simulated PCA conditions, the mixture of acetaminophen with six opioid drugs remained stable, maintaining at least 90% of its original concentration over 48 h. This stability is likely due to the slightly acidic pH of the mixed solution, which is close to the pH of acetaminophen injection, minimizing drug degradation. Consistent with the statistical results, there was no significant degradation in acetaminophen concentration within 48 h (P=0.12), supporting the overall stability of the admixtures. These findings are consistent with previous reports on the stabilizing effect of mannitol (10).

A slight increase in pH was observed in most of the mixtures within 48 h. This may be due to the buffering effect of mannitol in the acetaminophen-mannitol injection (pH 5.5-6.5), which slows the degradation process. However, the gradual release of alkaline metabolites, such as aminophenol breakdown products, could contribute to this slight pH increase. Additionally, the excipients or chemical properties of the different opioids may have influenced the pH of the solutions.

Preliminary experiments of our study revealed that the peak height measured based on the original concentration of the compatible solution exceeded the maximum peak height (5000 mAU) that could be determined using HPLC. Thus, the peak area could not be calculated. The entire peaks could be obtained after 2-32 times stepwise dilution with ultrapure water. Therefore, in this experiment, all test solutions related to peak area measurement were diluted 32 times with ultrapure water, except those used to determine appearance and pH.

Although the acetaminophen-mannitol injection showed stability within the 48-h timeframe, the compatibility and chemical stability of these drug mixtures beyond 48 h (25°C) require further investigation, particularly in clinical scenarios involving prolonged infusion or storage. Additionally, potential issues such as the formation of insoluble particles exceeding acceptable limits, as well as the effects of different wavelengths of light and temperature conditions on stability, remain to be explored. While the stability of acetaminophen was confirmed, it is not yet known whether acetaminophen-opioid combinations might affect the stability of the opioids (e.g., hydrolysis, oxidation).

Even though the experiment was conducted under *in vitro* conditions and may not fully reflect the stability of the drug in the complex *in vivo* environment, it still provided strong experimental evidence for the feasibility of combining acetaminophen with different analgesic drugs. Future studies should therefore focus on *in vivo* models, including liver cell experiments and animal studies, to further assess the safety of the acetaminophen-opioid combinations. These studies should aim to detect potential biomarkers of liver injury, such as alanine aminotransferase (ALT), to provide a more comprehensive understanding of the drug's safety profile in clinical settings.

## Conclusion

Acetaminophen-mannitol injection demonstrated good stability in terms of appearance, pH, and acetaminophen concentration when mixed with injections of morphine hydrochloride, sufentanil citrate, hydrocodone hydrochloride, pentazocine, butorphanol, and nalbuphine hydrochloride under natural light at 25°C within 48 h.

## Data Availability

The datasets used and/or analyzed during the current study are available from the corresponding author on reasonable request.
